# Lessons learned through piloting a community-based SMS referral system for common mental health disorders used by female community health volunteers in rural Nepal

**DOI:** 10.1186/s13104-020-05148-5

**Published:** 2020-07-01

**Authors:** Anvita Bhardwaj, Prasansa Subba, Sauharda Rai, Chaya Bhat, Renasha Ghimire, Mark J. D. Jordans, Eric Green, Lavanya Vasudevan, Brandon A. Kohrt

**Affiliations:** 1 Department of Population Family and Reproductive Health, Johns Hopkins Bloomberg School of Public Health, Baltimore, MD USA; 2 Duke Global Health Institute, Duke University, Durham, NC USA; 3 Transcultural Psychosocial Organization (TPO) Nepal, Baluwatar, Kathmandu, Nepal; 4 United Mission to Nepal, Kathmandu, Nepal; 5 Henry M Jackson School of International Studies and Department of Global Health, University of Washington, Seattle, WA USA; 6 Center for Global Mental Health, Institute of Psychiatry, Psychology and Neuroscience, Kings College London, London, UK; 7 Center for Health Policy and Inequalities Research, Duke Global Health Institute, Durham, NC USA; 8 Department of Family Medicine and Community Health, Duke University School of Medicine, Durham, NC USA; 9 Department of Psychiatry and Behavioral Sciences, George Washington University, Washington, DC USA

**Keywords:** Nepal, Developing countries, mHealth, Mental health, Help-seeking, Referral, Case-finding

## Abstract

**Objective:**

The Community Informant Detection Tool (CIDT) is a paper-based proactive case detection strategy with evidence for improving help-seeking behavior for mental healthcare. Key implementation barriers for the paper-based CIDT include delayed reporting of cases and lack of active follow up. We used mobile phones and structured text messages to improve timeliness of case reporting, encouraging follow up, and case record keeping. 36 female community health volunteers piloted this mobile phone CIDT (mCIDT) for three months in 2017 in rural Nepal.

**Results:**

Only 8 cases were identified by health volunteers using mCIDT, and only two of these cases engaged with health services post-referral. Accuracy with the mCIDT was considerably lower than paper-based CIDT, especially among older health volunteers, those with lower education, and those having difficulties sending text messages. Qualitative findings revealed implementation challenges including cases not following through on referrals due to perceived lack of staff at health facilities, assumptions among health volunteers that all earthquake-related mental health needs had been met, and lack of financial incentives for use of mCIDT. Based on study findings, we provide 5 recommendations—in particular attitudinal and system preparedness changes—to effectively introduce new mental healthcare technology in low resource health systems.

## Introduction

Mobile health (mHealth) has the potential to improve mental health in low- and middle-income countries (LMICs) by increasing awareness of and access to treatment [1–4]. The Community Informant Detection Tool (CIDT) was developed by Transcultural Psychosocial Organization Nepal (TPO-Nepal), under the Programme for Improving Mental Health Care (PRIME) [5, 6] to facilitate detection of and help-seeking for mental illness at the community level. The validated tool is a paper-based form consisting of vignettes of common symptoms using local idioms and illustrations [7–9]. The CIDT works by training trusted people in the community to detect people who match the vignettes and encourage them to seek care from mental health trained facility-based health workers (HW). In a pragmatic trial, use of paper-based CIDT was associated with 47% greater mental health treatment initiation compared to referral as usual [10].

Challenges in CIDT implementation have been identified, such as communication gaps between FCHVs and HW along with poorly maintained outpatient logs of CIDT referrals [8]. To address this, we designed a structure Short Messaging Service (SMS), i.e., text messaging, referral system to complement the paper-based CIDT process. This aimed to (i) reduce the communication gap between FCHVs and HWs at health posts, (ii) digitize the referral process and (iii) maintain online documentation. Our goals were to increase the rate of help-seeking, the number of people initiating care after being referred, and facilitating active follow-up from FCHVs.

## Methods

### Setting

This study was conducted in Nepal in 2017, 2 years after a 7.8 magnitude earthquake that saw an increase in mental illnesses in affected regions. The study site, 4 village development committees (VDCs) in Sindhuli district, was an earthquake affected region that had received immediate post-earthquake mental health services sponsored by an international organization and was now transitioning to government-funded operations. The introduction of the mCIDT was to foster sustaining and scaling of mental health referrals for this government transition.

### Development of mCIDT

**Fig. 1:**
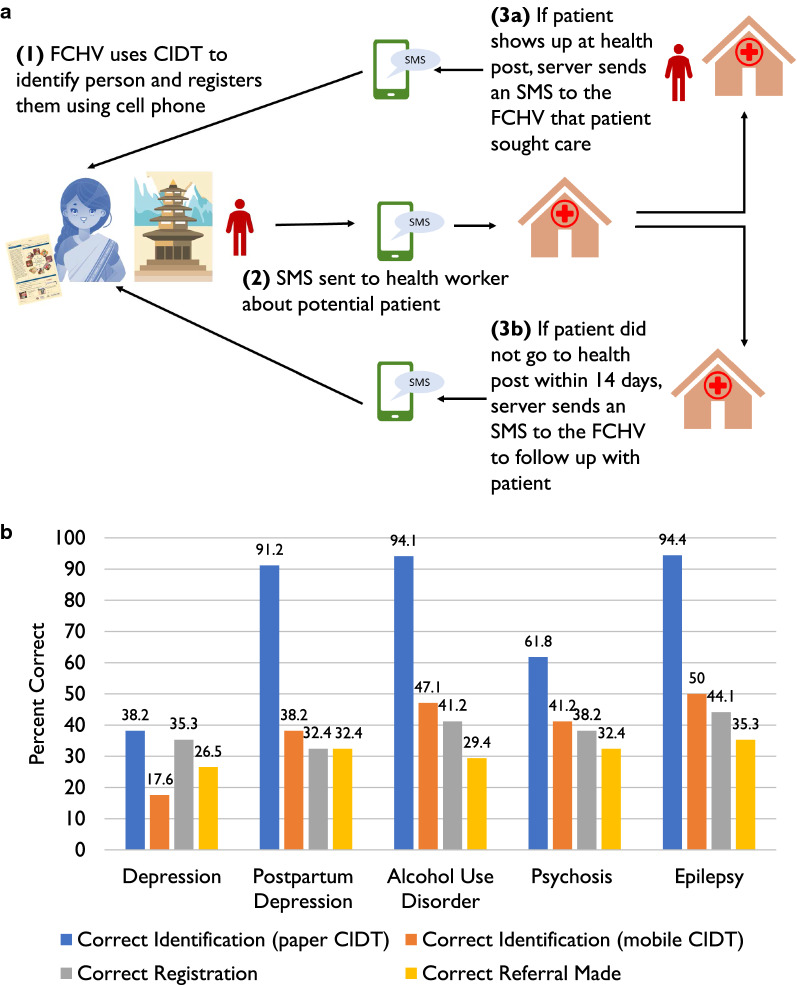
**a** mCIDT workflow; *CIDT* Community Informant Detection Tool, *FCHV* Female Community Health Volunteer, *SMS* Short Messaging Service. **b** Simulation data, performance of FCHVs (n = 34) for correct diagnosis using paper based CIDT compared to mobile CIDT

Due to the widespread use of standard feature phones (i.e., not smartphones), an SMS approach was selected for digitizing CIDT. An iterative process was used to develop and finalize the workflow for the SMS system. Digitization of the referral component of the CIDT was selected as the high value target to achieve this and a workflow was drafted (Fig. 1a). Because we only digitized the referral mechanism, the FCHVs continued to use the paper-based CIDT for detection of people with potential mental illness. Based on our previous CIDT experience, we estimated that one week would be an appropriate time window to visit the health facility once referred by the FCHV. If the person visited the health facility, s/he was registered in the system as a “complete case”, otherwise a reminder SMS was sent to the FCHV to follow up.

### Project implementation

A 3-day training for the FCHVs and HWs was conducted in July 2017. The training included use of basic mobile phone functions, role-playing using mCIDT, and a review of conditions included in CIDT. A codebook describing basic phone functions and the steps to refer using mCIDT was given to each FCHV for reference. After the training, we conducted 4 focus group discussions (FGD) with 36 FCHVs, 8 key informant interviews (KIIs) with HW, 2 KIIs with mental health experts, and 1 KII with an mHealth expert.

After the training, the mCIDT platform was implemented from August-October 2017. Supervision visits were completed twice at the health post at each VDC by the community clinical supervisor. In response to the lower than expected referrals seen near the end of the third month, a simulation workshop was held to re-train the FCHVs. A written vignette describing a mock case was read out loud to the FCHVs, who were asked to identify the case using CIDT. FCHVs were asked to use the mCIDT platform to refer the mock case (See Additional file 1: Figure S1). These steps were completed for a total of five vignettes addressing depression, postpartum depression, alcohol use disorder (AUD), psychosis, and epilepsy.

## Results

Demographics of the 36 FCHVs trained on mCIDT are included in Additional file 2: Table S1. Of these 36 FCHVs, only 8 FCHVs successfully implemented mCIDT, defined as referring someone. Several error messages including typos, missing spaces, wrong disorder codes, and incorrect sequencing were recorded in the system (Additional file 3: Table S2). Over 3 months of implementation, 8 FCHVs registered and referred 8 cases through mCIDT: 4 depression, 2 psychosis, 1 epilepsy, and 1 antenatal depression. No FCHV registered more than 1 case in the study period. Of those 8 cases who were referred, 2 cases visited the health facility, 2 could not be contacted in the follow-up. Four cases stated they did not seek treatment because the government health facility was not staffed with health workers.

After piloting the technology for 3 months, a simulation exercise was held with the FCHVs (n = 34) to determine their accuracy of using mCIDT (Fig. 1b). Level of education was significantly higher and age was significantly lower for the FCHVs who were able to correctly use mCIDT in comparison to those who were not able (Additional file 4: Table S3). Those who self-reported the ability to send an SMS and use the mCIDT Codebook were significantly more likely to correctly use mCIDT across all disorders.

Qualitative analysis of the KIIs, FGDs, field notes and observations by the research team elucidated the benefits of mCIDT, challenges, and recommendations to improve the program (See sample quotes from KIIs and FGDs in Table 1).

**Table 1:** Sample quotes from interviewees regarding use of mobile health Community Informant Detection Tool (mCIDT)

Theme	Description	Quote
Perceived benefits	Reduced travel burden for FCHVs when using mobile phone SMS for mCIDT	“*We don't have to go to the hospital time and again to ask about a patient. We don't have to ask the health worker. Previously we had to walk for 1 or 2 *h *to reach the health institution but we can know about them if we send the message from our home. We can also find if the patient has gone for the treatment or not through messages.” – FCHV in FGD* “*We got to know whether the patient went to hospital or not within a week after we had sent message despite the distance of patient's location. Otherwise, we need to go to the hospital to know if the patient went there or not. Now it is easy for us to go to patient’s home twice and ask about not going to the hospital.”— FCHV in FGD* * “FCHVS are engaged in other programs such as Vitamin A distribution, visiting the pregnant and recent mothers and were very busy in these activities due to which they had neglected mental health initially. But later when they were oriented about the mobile, they had a sense that they should work on this otherwise.”—FCHV in FGD*
Feasibility	Lack of feasibility for implementing mCIDT because FCHVs are overburdened	*“Because recently what we have been doing is the government has been mobilizing the FCHVs and we can see that FCHVs has been mobilized a lot. Because they have been engaging in programs related to maternal health, related to child health, and population statistics. And they have also been providing services regarding distribution of hygiene issues and home infestations so they’re quite busy so many times we have seen that they have not been referring the cases using CIDT due to the fact that they’re over-engaged and they have not been provided basic salary. Due to this fact I think if we can use other people, like teachers, or local clubs, or mothers’ groups I think there are a lot of mothers’ groups in the community, if we can mobilize them, we can better provide coverage to a large number of people. The FCHVs are overly busy with their schedule so if we can ease the burden of FCHVs on part and shift it to people to mothers group, teachers, and local leaders who have recently been elected, I think that can cause a huge impact and we might be getting a large number of referrals.”— KII with Mental Health Expert*
Lack of perceived need	Some FCHVs did not see mental health care as a need for their communities	*“There aren’t many cases in my ward. I cannot register anyone who doesn’t have problem.”— FCHV in FGD*
Stigma	Inability to use mCIDT because of mental health stigma	*“When people hear the word* manasik (*mental), they feel different. They don't want to engage at all. May be because of such stigma in the community, the FCHVs might have had problems.”— KII with Government Health Official*
Perceived difficulty of mental health care	Reluctance to work with mental health patients because of perceived difficulties	“*It’s difficult to work with* manasik samasya (*mental problems) It's easy to work on other areas but for mental problems, it's quite difficult.”—FCHV in FGD*
Privacy concerns	Reluctance to use mobile phones for mental health information	“*We need to create public awareness. Some patient has feeling that their illness is recorded in the phone and that information will be given to someone else.”—FCHV in FGD*
Low technological literacy	Inability to use SMS function on mobile phones	*“Some of the FCHVs were finding it difficult to use the mobile phones and using the menu key.” –KII with Mental Health CIDT Trainer* “*Another challenge would be difficulty in typing. We don't know how to type messages here. If it had been hand written, we could have written down some according to our capability but it is difficult to type it in the mobile.”—FCHV in FGD*
Supervision needs	Recommendation from FCHVs for more regular supervision when introducing technology	*“You taught them today, and when you call them after a month, they will get embarrassed if they are not able to do it. They might think that you'll scold them if they can't do it. Because of that fear, they will learn by whatever way they can e.g. by asking children, or looking at books, and come. But, if you leave as it is, then they might not care about it. Even if you don't scold later, if you keep following up with them from time to time, they might feel that they will be embarrassed, which will urge them to learn. I think the monthly supervision will be very beneficial.”— FCHV in FGD*

### Acceptability, feasibility and benefits

The FCHVs, HWs and mental health experts, thought the greatest benefit of mCIDT could be reducing the burden of work on FCHVs. Secondary benefits included the potential for better communication between HWs and FCHVs. Mental health and mHealth experts were wary of the FCHVs ability to use the mobile phones and stated that maintaining patient confidentiality was not feasible.

### Challenges

The simulation results showed that most FCHVs were unable to utilize mCIDT, this challenge was evident in the interviews as well. We summarized these challenges in 5 domains: Community, Participant, Facility, Program, and Technological.

The most prominent challenges were mentioned at the community level. FCHVs repeatedly said no mental health cases were present in the community, which is inconsistent with assessments finding high rates of mental health and psychosocial problems in the area [11]. This pointed to a lack of community awareness of the burden of mental illnesses. FCHVs mentioned that previously Home-Based Community Workers (HBCW) in the area were responsible for identification of mental health cases, and they had been paid by an international organization to implement CIDT after the earthquake.

FCHVs acknowledged that stigma towards mental health is persistent in the community. If an FCHV identified someone with a potential mental illness, it was difficult to gain support from the family to get the patient to care. FCHVs were aware that AUD cases resided in the communities, but they were uncomfortable interacting with the patient fearing he was violent or thinking the patient cannot get better. FCHVs particularly felt discomfort dealing with male patients citing their gender roles. The fact that these FCHVs lived in the same community and they did not want to have potential conflict also contributed. FCHVs said that community members were worried about breaches of confidentiality due to the use of a mobile phone.

FCHVs struggled to use the mobile phone for reasons ranging from poor eyesight among older FCHVs to lack of confidence using technology. Lack of technological literacy was the most frequent issue observed during training sessions. It was also noted by trainers that the need to focus on how to use a mobile phone was unanticipated. Lower education became a barrier when trying to type and send the structured SMS. A major challenge was transferring the visual information on paper-based CIDTs into appropriate syntax for the structured SMS. Lower education also became an issue when receiving error messages and the inability to read and respond with the correction. Interviewees, who were not FCHVs, discussed government challenges in the context of implementing a policy that would set an educational threshold for FCHVs.

FCHVs are also overburdened through engagement in many parts of the health sector. Absenteeism of HW at the health post discouraged one FCHV whose referred case had to return without services. Financial incentives were brought up by most FCHVs as a way to increase motivation for them to engage in the mental health sector. Network instability was one technological challenge.

### Suggested recommendations from participants

The main recommendations centered around more supervision for the FCHVs and increasing the level of awareness about mental health in the community.

## Discussion

mCIDT was designed as a tool to strengthen the government’s mental health services by increasing the number of cases referred from the community for care-seeking. Piloting of the mCIDT platform for 3 months in 4 VDCs resulted in only 8 referrals. This equates to 0.67 mCIDT referrals per VDC per month, with a 25% treatment seeking rate. This is considerably lower than the 615 paper CIDT referrals with 364 seeking treatment in 24 VDCs during the 2-year post-earthquake period, which equates to 1 CIDT per VDC per month, with 60% treatment seeking rate [11]. Our prior studies in non-earthquake settings in Nepal also identified a 67% treatment seeking rate [9]. Qualitative and quantitative data from the study revealed numerous barriers and challenges with implementation of mCIDT. After this pilot period, mCIDT was not continued or scaled-up due to these challenges.

Although prior studies have highlighted technological barriers (e.g., lack of electricity and poor networks) as prominent barriers to mHealth in LMIC settings [12], we found that these were not among the most important barriers when introducing mCIDT as a novel mental health mHealth initiative in rural Nepal. In our study, lower educational levels, older age, and inability to send text messages were associated with incorrect use of mCIDT. This is consistent with systematic review of mHealth tools used by frontline health workers in LMICs that identified age, level of education, and years of experience of the health workers as primary barriers to adoption of mobile technologies [13]. Similarly, a study in rural Nigeria also showed that younger and more educated midwives demonstrated higher scores on knowledge assessments for mHealth technology use [14].

A striking lesson from our study was the lack of motivation to engage in mental health work. This is consistent with overall lack of importance placed upon mental health among health workers and stigmatization of providing mental health care [15]. Another challenge to motivation among FCHVs was the shift from CIDT work being compensated through an international organization in the immediate post-earthquake period to FCHVs expected to conduct their work, including CIDT, through the government system without pay after the earthquake relief phase.

## Limitations

With the mCIDT tool only piloted for 3-months, the study duration was relatively short and it is difficult to identify patterns with only 8 potential mental illness cases identified and only 2 seeking treatment after the referral. When the paper version was used in other areas, this was in the context of a broader district mental health plan to improve primary care services and community awareness [16]. We did not evaluate the community’s perceptions of the feasibility and acceptability of the tool prior to its implementation. In our other work with introduction of mHealth technologies in Nepal, we first conducted qualitative studies to identify acceptability and potential barriers to implementation [17].

Based on the experiences and challenges encountered in this study, we have provided recommendations for future studies introducing new technology in low resource settings to address mental health needs (see Table 2 for recommendations). Of particular importance, we recommend that any technological introduction for mental health services should be accompanied by approaches to also assure motivation to work with mental health patients [15]. This is crucial not only among community health volunteers using new technology but also throughout the health system to assure that there are health workers are motivated to treat newly identified mental health patients.

**Table 2:** Recommendations for introduction of novel technological applications for mental health care in low resource settings

Recommendation	Example
1. Conduct qualitative assessments prior to implementing new technology	Qualitative methods accompanied by demonstration of new technology can be used to determine acceptability, feasibility, and other potential implementation barriers
2. Consider how stigma related to mental illness impacts use of technology	In settings with high stigma against mental illness, consider how technology could be used to address anonymity and reduce risk of breaching confidentiality
3. Assure that adequate services are in place for mental health care	When piloting new technology, assure availability of basic mental health services to reduce risk of low adoption because of perceived lack of care/supportive human resources or infrastructure
4. Address perceived need for both care and technological solutions	Prior to implementation, explore community and health worker perceptions of need, and address these needs alongside technical skills in trainings
5. Explore multiple stakeholder groups for adopting technology	Given the potential for differential uptake of technology, pilot with multiple stakeholder groups (e.g., community health workers, teachers, community groups, religious leaders)

## Supplementary information

**Additional file 1: Figure S1** Steps for using mCIDT to refer patient.

**Additional file 2: Table S1** Demographics of Female Community Health Volunteers (n=36) trained in mCIDT platform.

**Additional file 3: Table S2** Types of messages stored in server.

**Additional file 4: Table S3a** Factors associated with correct and incorrect use of mCIDT for each diagnosis, categorical variables (n = 34); **b** Factors associated with correct and incorrect use of mCIDT for each diagnosis, continuous variables (n = 34).

## Data Availability

The datasets analyzed during the current study are available from the corresponding author on reasonable request.

## Abbreviations

CIDT: Community Informant Detection Tool
FCHV: Female community health volunteer
mHealth: Mobile health
mCIDT: Mobile health community informant detection tool
TPO Nepal: Transcultural Psychosocial Organization Nepal
LMIC: Low- and middle-income countries
PRIME: Programme for Improving Mental Health Care
SMS: Short messaging service
HBCW: Home based care worker
HW: Health workers
AUD: Alcohol use disorder
PTSD: Post-traumatic stress disorder
IRB: Institutional review board
VDC: Village Development Committee
