# Supplementary material for: Lessons learned through piloting a community-based SMS referral system for common mental health disorders used by female community health volunteers in rural Nepal
Source: BMC Res Notes. 2020 Jul 1;13:309. doi: 10.1186/s13104-020-05148-5 (PMC7328268; doi:10.1186/s13104-020-05148-5)
Supplement: Supplementary file 1 — Additional file 1: Figure S1 Steps for using mCIDT to refer patient. [file 13104_2020_5148_MOESM1_ESM.pdf]

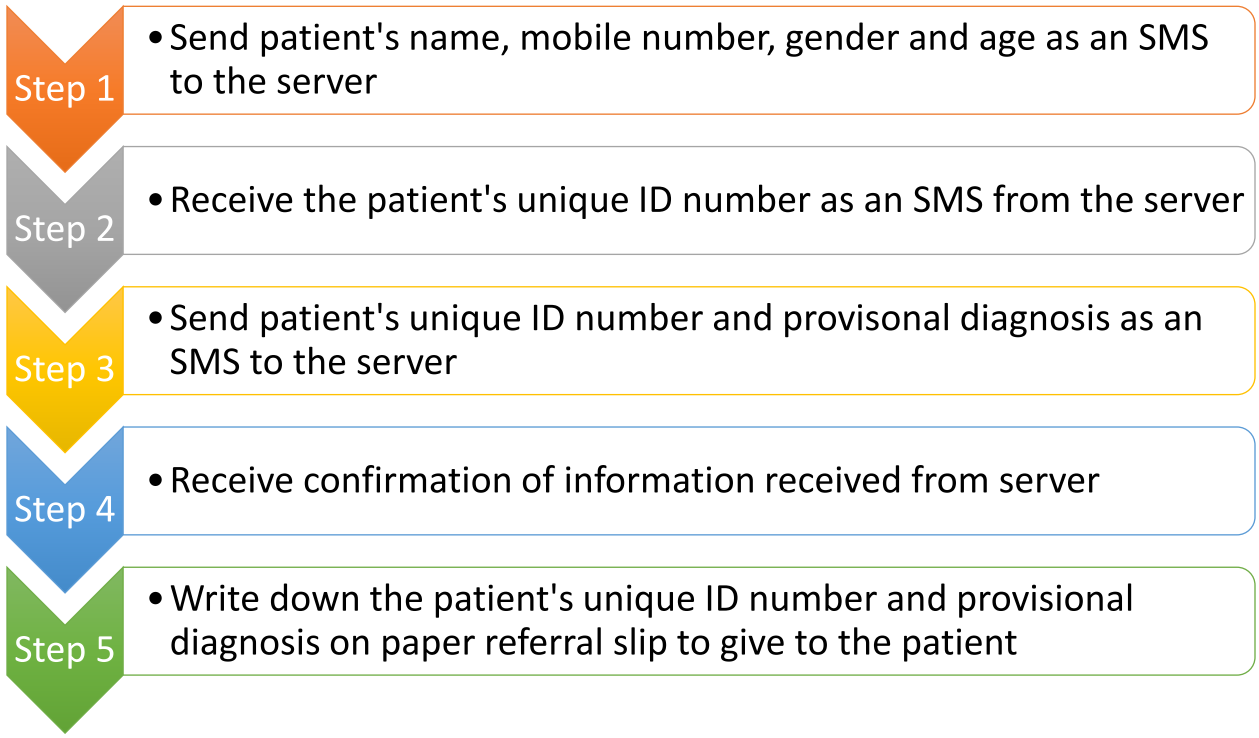
Step 1
Send patient's name, mobile number, gender and age as an SMS to the server
Step 2
Receive the patient's unique ID number as an SMS from the server
Step 3
Send patient's unique ID number and provisonal diagnosis as an SMS to the server
Step 4
Receive confirmation of information received from server
Step 5
Write down the patient's unique ID number and provisional diagnosis on paper referral slip to give to the patient
