# Supplementary material for: Lessons learned through piloting a community-based SMS referral system for common mental health disorders used by female community health volunteers in rural Nepal
Source: BMC Res Notes. 2020 Jul 1;13:309. doi: 10.1186/s13104-020-05148-5 (PMC7328268; doi:10.1186/s13104-020-05148-5)
Supplement: Supplementary file 2 — Additional file 2: Table S1 Demographics of Female Community Health Volunteers (n=36) trained in mCIDT platform. [file 13104_2020_5148_MOESM2_ESM.doc]

**Additional File 2: Table S1.** Demographics of Female Community Health Volunteers (n=36) trained in mCIDT platform

| **Variable** | **N** | **(%)** |
| --- | --- | --- |
| Age (in years) |  |  |
| 18-25 | 0 | 0 |
| 26-35 | 5 | 13.8 |
| 36-45 | 9 | 25 |
| 46-55 | 11 | 30.5 |
| 56+ | 11 | 30.5 |
| Education |  |  |
| Illiterate | 4 | 11.1 |
| Literate (no formal education) * | 20 | 55.6 |
| Primary (1-5^th^ Grade) | 3 | 8.3 |
| Grade 5-10 | 4 | 11.1 |
| SLC pass or above | 5 | 13.9 |
| Health Catchment Area |  |  |
| Village Development Committee A | 9 | 25 |
| Village Development Committee B | 9 | 25 |
| Village Development Committee C | 9 | 25 |
| Village Development Committee D | 9 | 25 |

*****Literate defined as a female community health volunteer who is able to read and write her name
