# Supplementary material for: Lessons learned through piloting a community-based SMS referral system for common mental health disorders used by female community health volunteers in rural Nepal
Source: BMC Res Notes. 2020 Jul 1;13:309. doi: 10.1186/s13104-020-05148-5 (PMC7328268; doi:10.1186/s13104-020-05148-5)

**Additional file 3: Table S2. Types of messages stored in server**

**
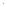
**

| **Message Type** | **Tally** |
| --- | --- |
| Command prompts | 21 |
| Confirmation prompts | 23 |
| Wrong answer (wrong syntax) | 18 |
| Disease/ condition code incorrect | 2 |
| Wrong phone number | 3 |
| Wrong patient name | 1 |
| System error message | 6 |


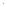

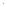

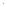

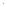

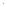

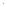

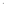

Supplement: Supplementary file 3 — Additional file 3: Table S2 Types of messages stored in server. [file 13104_2020_5148_MOESM3_ESM.doc]
