# Supplementary material for: Lessons learned through piloting a community-based SMS referral system for common mental health disorders used by female community health volunteers in rural Nepal
Source: BMC Res Notes. 2020 Jul 1;13:309. doi: 10.1186/s13104-020-05148-5 (PMC7328268; doi:10.1186/s13104-020-05148-5)
Supplement: Supplementary file 4 — Additional file 4: Table S3a Factors associated with correct and incorrect use of mCIDT for each diagnosis, categorical variables (n = 34); b Factors associated with correct and incorrect use of mCIDT for each diagnosis, continuous variables (n = 34). [file 13104_2020_5148_MOESM4_ESM.pdf]

Table S3a. Factors associated with correct and incorrect use of mCIDT for each diagnosis, categorical variables (n=34)

	**Psychosis**			**Alcohol Use Disorder**			**Postpartum Depression**			**Epilepsy**		
	Incorrect mCIDT, n (%)	Correct mCIDT, n (%)	*p*-value	Incorrect mCIDT, n (%)	Correct mCIDT, n (%)	*p*-value	Incorrect mCIDT, n (%)	Correct mCIDT, n (%)	*p*-value	Incorrect mCIDT, n (%)	Correct mCIDT, n (%)	*p*-value
**Education**			.000			.000			.000			.000
Illiterate	1(0)	3(100)		1(25)	3(75)		2(50)	2(50)		1(25)	3(75)	
Literate	17(89.5)	2(10.5)		17(89)	2(10)		17(89)	2(11)		16(84.2)	3(15.3)	
Grade 1-5	2(66.7)	1(33.3)		0(0)	3(100)		1(33.3)	2(66.7)		0(0)	3(100)	
Grade 5-10	0(0)	3(100)		0(0)	3(100)		1(33.3)	2(66.7)		0(0)	3(100)	
SLC pass & above	0(0)	5(100)		0(0)	5(100)		0(0)	5(100)		0(0)	5 (100)	
**Ward**			.267			.368			.184			.168
Ratanchura (1)	4(44.4)	5(55.6)		4(44.4)	5(55.6)		4(44.4)	5(55.6)		2(22.2)	7(77.8)	
Bhimeshwor (2)	4(44.4)	5(55.6)		3(33)	6(66)		4(44.4)	5(55.6)		4(44.4)	5(55.6)	
Bhuwaneshwori (3)	7(87.5)	1(12.5)		6(75)	2(25)		6(75)	2(25)		5(62.5)	3(37.5)	
Basehwor (4)	5(62.5)	3(37.5)		5(62.5)	3(37.5)		7(87.5)	1(12.5)		6(75)	2(25)	
**Can send a SMS**			.017			.003			.039			.015
No	13(81.2)	3(18.7)		13(81)	3(19)		13(81)	3(19)		12(75)	4(25)	
Yes	7(38)	11(62)		5(27)	13(73)		8(44.4)	10(55.6)		5(27.8)	13(72.2)	
**Can use mCIDT Codebook**			.004			.000			.012			.084
No	15(83.3)	3(16.7)		15(83)	3(17)		15(83)	3(17)		12(66.7)	6(33.3)	
Yes	5(31.3)	11(68.7)		3(18.8)	13(81.3)		6(37.5)	10(62.5)		5(31.3)	11(68.8)	

Two-tailed Fisher's exact test t-test compares correctly or incorrectly completing mCIDT step 1 message completion among persons who accurately identified paper CIDT.

Table S3b. Factors associated with correct and incorrect use of mCIDT for each diagnosis, continuous variables (n=34)

	Psychosis				Alcohol Use Disorder				Postpartum Depression				Epilepsy			
	Incorrect mCIDT, Median (IQR)	Correct mCIDT, Median (IQR)	Test statistic (Wilcoxon)	p-value	Incorrect mCIDT, Median (IQR)	Correct mCIDT, Median (IQR)	Test statistic (Wilcoxon)	p-value	Incorrect mCIDT, Median (IQR)	Correct mCIDT, Median (IQR)	Test statistic (Wilcoxon)	p-value	Incorrect mCIDT, Median (IQR)	Correct mCIDT, Median (IQR)	Test statistic (Wilcoxon)	p-value
**Age (years)**	57 (48, 60)	40 (35, 51.25)	17.50	.009	52.5 (47.25, 59.5)	42 (35, 48.75)	40.00	.001	51.5 (44.8, 60)	45 (36, 48.5)	57.00	.016	53 (46.5, 60.5)	45 (36, 52)	67.00	.08
